# Quantitative measurement of tympanic membrane structure and symmetry with optical coherence tomography in normal human subjects

**DOI:** 10.1117/1.JBO.30.5.056007

**Published:** 2025-05-14

**Authors:** Zihan Yang, Marcela Moran Mojica, Wihan Kim, John S. Oghalai, Brian E. Applegate

**Affiliations:** a University of Southern California, Caruso Department of Otolaryngology - Head and Neck Surgery, Los Angeles, California, United States; b University of Southern California, Alfred Mann Department of Biomedical Engineering, Los Angeles, California, United States; c University of Southern California, Ming Hsieh Department of Electrical and Computer Engineering, Los Angeles, California, United States

## Abstract

**Significance:**

Early detection of ear pathology is essential for preventing hearing loss, yet the sensitivity of otoscopic examinations by primary care providers during annual physicals remains low. Optical coherence tomography (OCT) offers a promising alternative for detailed imaging of the tympanic membrane (TM) and middle ear (ME), providing the potential for early identification of ear disease.

**Aim:**

We aim to develop a quantitative method for assessing symmetry between the right and left ears and to establish a baseline for this approach in normal subjects.

**Approach:**

Volumetric OCT images were acquired from 12 normal subjects using a custom hand-held OCT otoscope. A volume registration and fusion method was applied to expand the TM field of view, followed by TM thickness measurement and generation of 3D thickness maps. The symmetry between left and right TMs was quantitatively analyzed using the Dice similarity coefficient.

**Results:**

The average TM thickness was measured as 73.89±14.79  μm for left ears and 70.72±11.58  μm for right ears, with no statistically significant difference at the 0.05 level. The symmetry analysis revealed a mean similarity coefficient of 0.79±0.02 between left and right ears among the 12 normal subjects.

**Conclusions:**

OCT imaging enables quantitative assessment of TM thickness and symmetry, offering a baseline for identifying early ear pathologies.

## 1 Introduction

Early identification of ear disorders is important for effective and timely patient management.^1^ The traditional otoscope and surgical microscope, which serve as the main visualization tools for the tympanic membrane (TM) and middle ear (ME), have several limitations.^2^ Otoscopy can only provide visualization of the lateral surface of the TM. Deeper structures within the middle ear, such as the ossicles, are not well visualized, making diagnosing ME pathologies such as otitis media more challenging.^3^ Similarly, it is difficult to quantify the morphology in any standard way using otoscopy, contributing to the low diagnostic yield.

Other medical imaging technologies, such as computed tomography (CT) and magnetic resonance imaging (MRI), can readily quantify morphology but nevertheless are used sparingly for TM and ME pathologies because of their low contrast and poor resolution.^4^ CT captures bone abnormalities; however, it has poor contrast for soft tissue differences and carries the risk of radiation exposure.^5^ MRI has good contrast for soft tissues, but the spatial resolution is insufficient to clearly visualize ME structures and the images contain artifacts in patients with metallic middle ear ossicular implants.^6^ Ultrasound imaging, also commonly used in patients to study the abdomen, neck, or extremities, requires an acoustic couplant, typically a gel. This requirement poses a significant obstacle to *in vivo* imaging of the ear in the clinic.^7^

Optical coherence tomography (OCT) is a noninvasive imaging technique that allows for real-time, high-resolution imaging of microstructures and has been widely used in ophthalmology.^8^^,^^9^ In otology, OCT also has been shown to have promise for clinical imaging, leveraging its resolution, contrast, and imaging range.^10^^–^^14^ OCT imaging of the ME enables two-dimensional (2D) and three-dimensional (3D) visualization of TM and ME structures.^15^^–^^17^ Some studies have focused on structural or functional imaging of *ex vivo* temporal bone. Jang et al. visualized the TM thickness by employing spectral domain OCT (SDOCT) to evaluate the effectiveness of an experimental TM repair scaffold in guinea pigs with chronic TM perforation.^18^ Van der Jeught et al. measured the thickness distribution of the TM of six human temporal bones using SDOCT with a large field of view.^19^ Chang et al. used OCT to view the sound-induced motion of the TM and ossicles of the chinchilla ear simultaneously.^20^ The preliminary study from Park et al. on cadaveric human temporal bone models evaluated the vibration of the TM and ossicles with sub-nanometer resolution.^21^ Burkhardt et al. employed Doppler OCT to investigate the oscillatory behavior of the TM surface of a fresh excised human temporal bone.^22^

Equipped with a handheld otologic probe, OCT enables imaging of the TM and ME anatomy *in vivo.*^23^ OCT imaging of the TM has been used for the diagnosis of otitis media (OM), where chronic OM can be diagnosed by the detection of a biofilm behind the TM.^24^^,^^25^ In addition, previous studies have demonstrated TM thickness as a proposed metric to differentiate normal, chronic, and acute OM infections.^15^^,^^26^^–^^28^ Another related application is to identify middle ear effusions,^29^ which was achieved with an accuracy of 90.6%.^30^ In addition, OCT has been shown to be capable of differentiating cholesteatoma from normal ME mucosa.^31^^,^^32^ Although OCT has been used to study these various pathologies, there is also a need to understand and quantify the normal ear.

Knowledge of ear symmetry has been used to understand the structural development of children’s ears, evaluate early otologic lesions, and even design hearing aid instruments. Fu et al. evaluated the morphological variations of the entire 3D external ear with parameterized 3D ear modeling.^33^ Claes et al. investigated the matching symmetry in anatomical substructures of the human pinnae using 3D spatially dense geometric morphometrics, which have important implications for ear recognition and sound localization.^34^ Alexander et al. examined variations in the shape of the human ear according to age, sex, and ethnic group and found that most linear measurements including height and width between left and right ears were highly symmetrical.^35^ Although previous studies have reported the position and symmetry of external ear dimensions, the symmetry in the middle ear has not yet been studied.^36^^–^^38^ Inspired by studies on the symmetry of the external ear, the symmetry of the middle ears may be effective for early diagnosis of ME pathologies. Although OCT has been used in the study of TM and ME, there has been no quantitative comparison of the symmetry of left and right ears. Quantitative measures of symmetry could enable various applications, including monitoring progressive diseases and early diagnosis of pathologies.

This study performed a quantitative analysis of the left and right ears in 12 normal subjects. Multiple OCT volumes were acquired using a custom hand-held otologic OCT (HHOCT) system integrated with an additional endoscopic camera. To increase the TM imaging range, an optimized volume registration strategy was developed, and an OCT volume stitching method was employed. Finally, the thickness and the symmetry of TM between the left and right ears were quantitatively evaluated based on registered OCT volumes.

## 2 Methods

A custom HHOCT system was developed for TM and ME imaging and reported in our group’s previous study.^39^ The system is built on a medical cart and includes the HHOCT imaging probe, the optical fiber-based OCT interferometer, the sub-electronic devices for data processing, and a foot pedal for data collection assistance. A swept source laser employed by the OCT system has a central wavelength of 1310 nm, a bandwidth of 39 nm, and a sweep rate of 200 kHz, enabling rapid scanning of ME morphological structures in a clinical setting. The system is capable of achieving a $7.4\times 7.4\;{\mathrm{mm}}^{2}$ field of view (FOV) at the focal plane of the objective with a Nyquist imaging depth of 18.3 mm, lateral resolution of 38  μm, and axial resolution of 33.4  μm (in tissue, n=1.3). The HHOCT imaging probe also equips a custom on-device otoscopic camera system, enabling simultaneous video otoscopy with OCT imaging, as shown in Fig. S1 in the Supplementary Material.

In our study, 12 normal subjects (five males and seven females) were imaged using the HHOCT system (Table S1 in the Supplementary Material). Multiple OCT volumetric images were collected for each subject’s ear. The co-registered integrated video otoscopy allowed for the OCT FOV to be precisely moved to different areas of the TM. The total imaging time per session, including consenting the subject and explaining results, is less than 5 min. This study was approved by the Institutional Review Board (IRB) at the University of Southern California (USC, IRB: HS-17-01014).

**Fig. 1:**
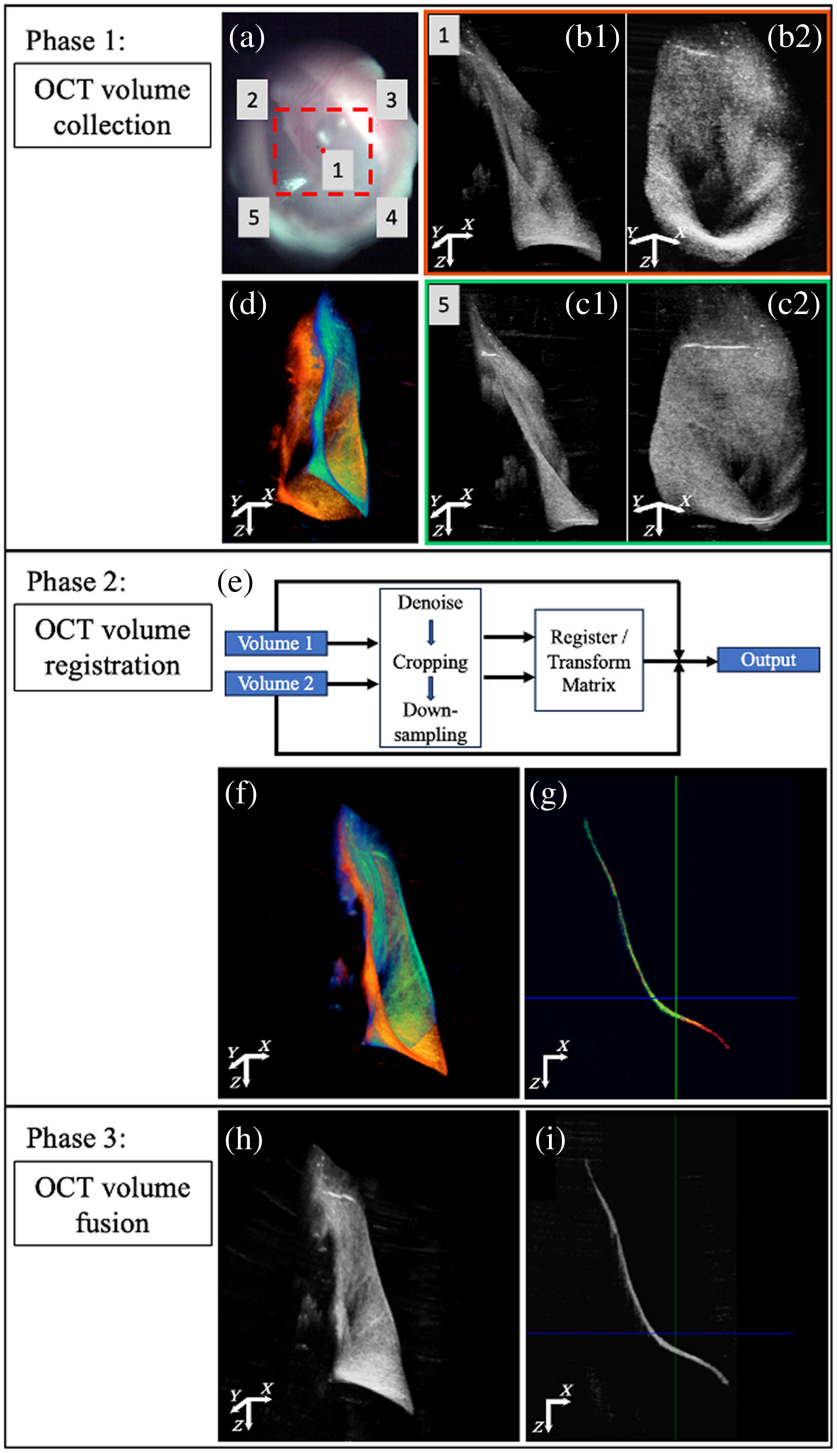
Workflow of OCT volume stitching algorithm. (a)–(d) OCT volume collection protocol. (e)–(g) OCT volume registration. (h) and (i) OCT volume fusion.

To achieve a larger imaging area than the system’s FOV, we employed a volume stitching algorithm. This allowed us to produce a volume image that included all the TM and ME structures viewable through the ear canal. The workflow is divided into three phases, as shown in Fig. 1. Phase 1 involves the collection of OCT volumes at multiple positions to cover the desired FOV. The acquisition protocol is illustrated in Fig. 1(a). Using our HHOCT probe, which captures otoscopic and OCT images simultaneously, we identified five positions on the TM for acquiring volumetric OCT images. These positions provided significant overlap, facilitating registration during post-processing. Each volume took ∼1.5  s to acquire. Figures 1(b1) and 1(b2) show the OCT volume at position 1, whereas Figs. 1(c1) and 1(c2) show the OCT volume at position 5 with two different orientations, respectively.

**Fig. 2:**
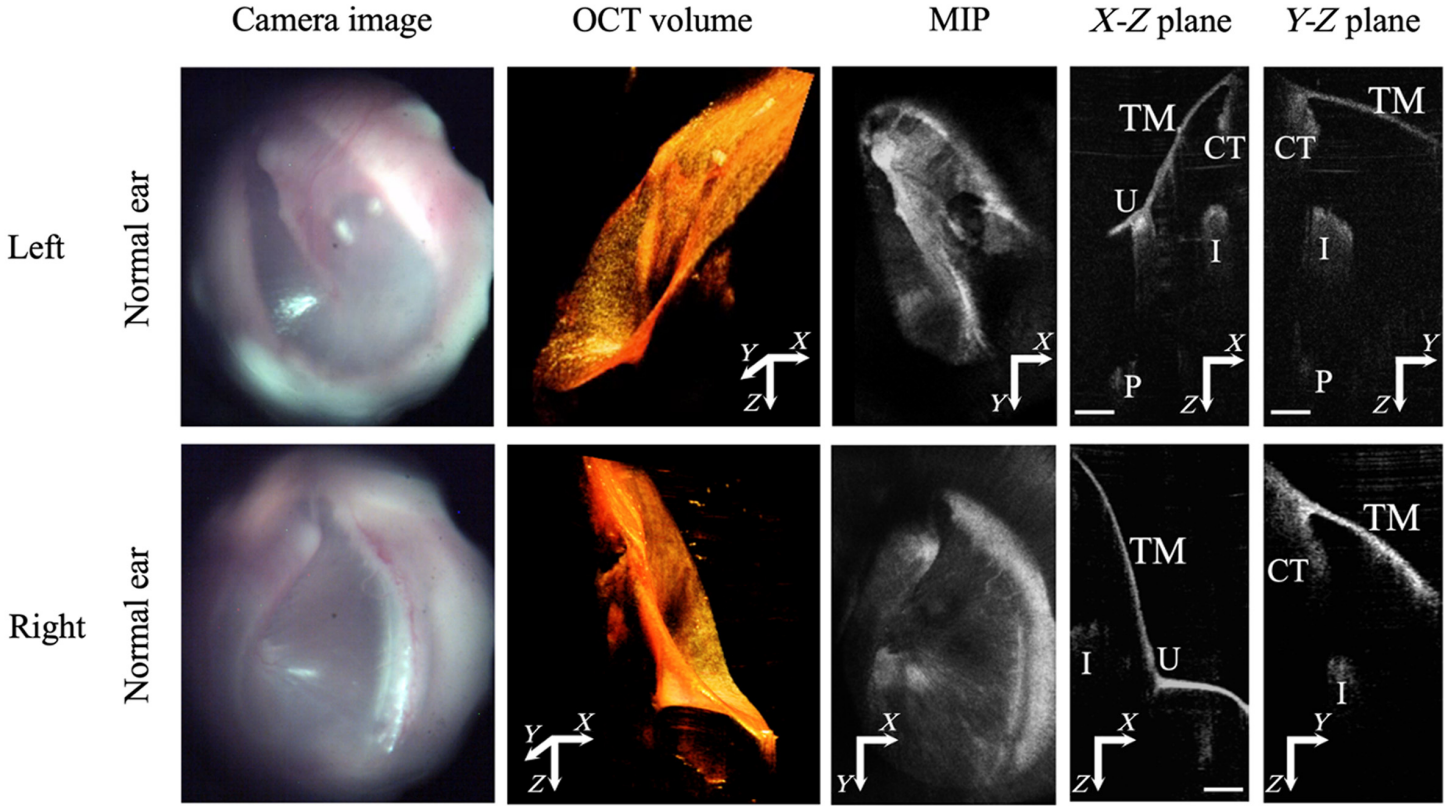
Otoscopic images and corresponding OCT volume images, mean intensity projection (MIP), and cross-sections, obtained from the OCT otoscope. Scale bar: 1 mm. CT: Chorda tympani. I: Incus. P: Promontory. U: Umbo. TM: Tympanic membrane.

Due to the anatomical variations in each subject’s ear canal shape, the HHOCT imaging probe must be repositioned to provide a clear view and ensure the subject’s comfort. This repositioning involves rotating the probe to the subject’s ear canal, making simple translation-based registration insufficient, as illustrated in Fig. 1(d), where the volumes are displayed using orange-red and blue-green colormaps. In phase 2, we used an OCT volume registration method based on a 2D registration across multiple planes within the volume. To reduce registration time and improve accuracy, image denoising and cropping are applied to remove the background. In addition, we also down-sampled the data to improve processing speed, selecting 4× down-sampling rate to balance registration accuracy and computational time [Fig. 1(e)].

In 3D space, the *en face* images (XY plane) for each pair of two OCT volumes are first registered. Subsequently, two separate registrations were performed in XZ and YZ planes. The three resulting transformation matrices were then combined into the final transformation matrix and applied to the original (non-down-sampled) images. As shown in Fig. 1(f), the OCT volumes at positions 1 and 5 are matched very well. A cross-section of the co-registered volumes is presented in Fig 1(g) to further illustrate the quality of the registration. This process was repeated for the remaining collected volumes, registering each with the volume in position 1.

To maintain continuity and consistency in the registered images, we performed OCT volume fusion in phase 3 after all volumes had been registered. The intensity values of each corresponding pixel in the overlapping areas of the registered OCT volumes were summed and averaged. The final result for this volume set is shown in Figs. 1(h) and 1(i).

Segmentation of the TM is a key preprocessing step for all the analyses presented below. It was accomplished by connected component labeling of binary OCT images. The fused OCT images are first binarized and then undergo connected component labeling to extract the TM. If necessary, fine-tuning is performed by manual segmentation.

Calculating the thickness of the roughly conical-shaped TM is nontrivial. At every point on the TM, we must compensate for the relative angle of the surface. To accomplish this, we used the following algorithm to derive a TM thickness map from the fused OCT volume image, similar to the method we have described previously.^23^ First, we subtract the background-volume image without a sample to remove the system’s artifact. Next, we apply a mean filter along the depth axis to remove the saturation artifacts. We generate a binary mask by applying a 3D median filter and utilizing a threshold based on Otsu’s algorithm.^40^ Subsequently, we measure the TM thickness using the minimum line integral method, an efficient technique developed for extracting tissue thickness in MRI.^41^ This method calculates line integrals passing through the center points in the binary mask and selects the minimum integral value as the thickness of this position. The algorithm outputs both a thickness map and the direction of the minimum line integral, which quantifies the complex shape of the TM.

The first step in analyzing the symmetry between the right and left ears is to co-register the volumetric images after inverting the left ear image. We employed the same registration algorithm as described above to calculate a transformation matrix for this purpose. The transformation matrix is then applied to the original volumetric data, aligning the left ear with the right ear of each subject.

After co-registering the right and left ears, we applied a measurement method based on the Dice similarity coefficient (DSC) to evaluate their symmetry. The DSC ranges from 0 to 1, where a DSC value of 1 indicates a perfect overlap between the left and right ears. As shown in Eq. (1), the $n_{\mathrm{th}}$ DSC is given by

$$\text{Dice}(A_{L},A_{R}{)}_{n}=\frac{2(A_{L,n}\cap A_{R,n})}{A_{L,n}\cup A_{R,n}},$$ (1)

where the DSC is defined as the ratio of the set of pixels (A) to the total set of pixels. The DSC is calculated for each B-scan; hence, the first step is to sequentially extract co-registered B-scans from the two volumes. Next, a binarization process is performed, setting the background to 0 and any signal from the TM to 1. Finally, volume symmetry is characterized by the mean and standard deviation of the Dice values.

The mean and standard deviation of TM thickness for each ear are calculated. The TM thickness for each pair of ears (left and right) is compared using a two-tailed t-test, with a p-value <0.05 was considered statistically significant. Similarly, TM thickness and symmetry were statistically analyzed separately for female and male subjects.

## 3 Results

Both the left and right ears of 12 normal subjects were imaged using the custom HHOCT otoscope. Representative results are shown in Fig. 2, where the top row shows the images of the left ear, and the bottom row shows the images of the right ear of a single patient. The first column shows the otoscopic images, similar in quality to what would be collected from a commercial otoscope. The second to fifth columns are OCT images acquired simultaneously using the same hand-held probe. The second column shows the reconstructed OCT volumes, which clearly display the TM and ME structures behind the TM. The third column shows the mean intensity projection (MIP) of the OCT volumes, where the mean is in the depth dimension. The MIP images show similar features to the otoscope image in the 1st column, which allows us to confirm the correct orientation and relative scaling of the OCT volume relative to the otoscopic image. The fourth and fifth columns show OCT images in two orthogonal planes taken from the OCT volumes. The TM and ME structures, including the umbo (U), incus (I), chorda tympani (CT), and cochlear promontory (P), can be clearly observed.

**Fig. 3:**
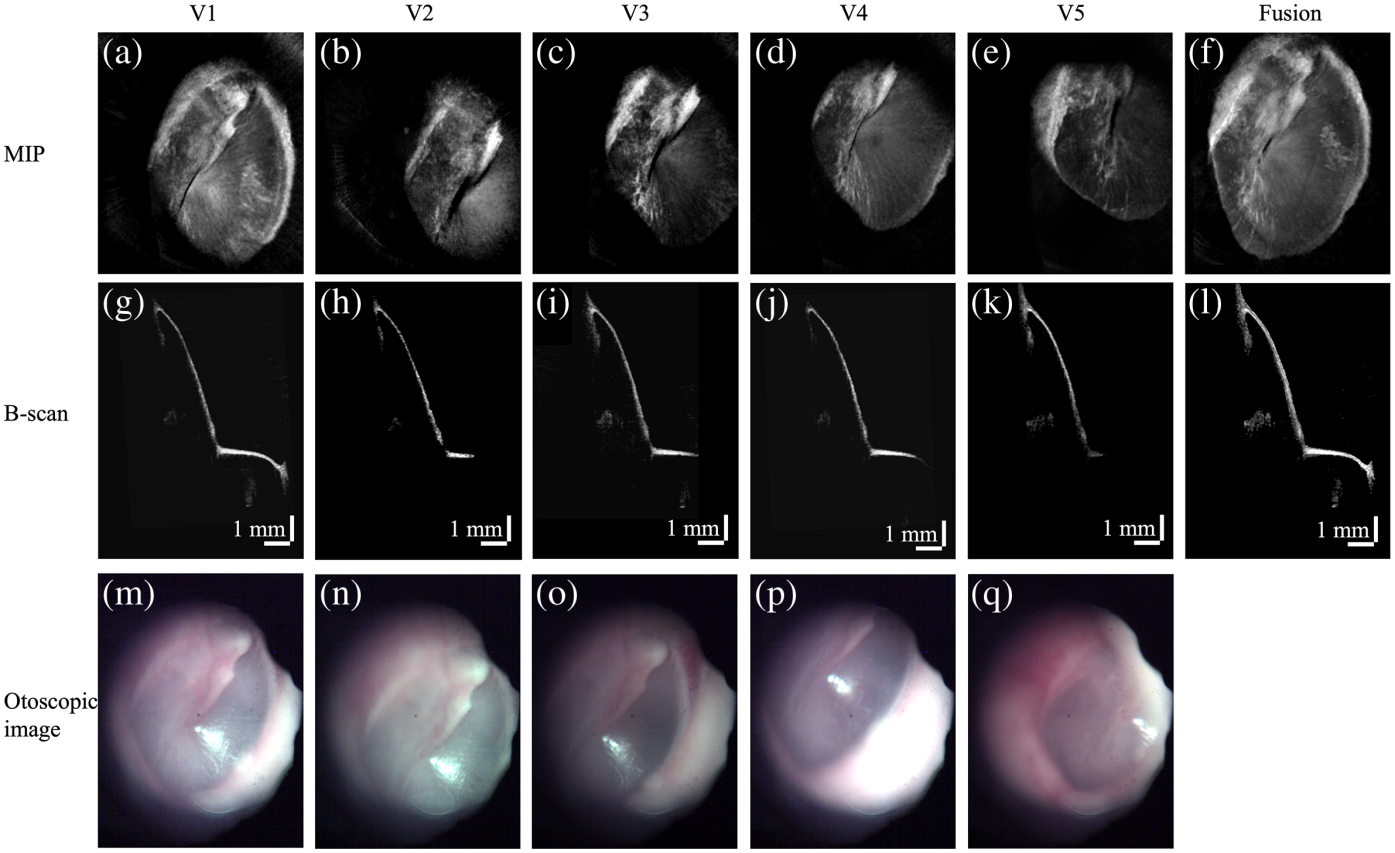
Representative result of OCT volume stitching. (a)–(e) MIP images of OCT volumes at five different locations. (g)–(k) OCT B-scans of registered OCT volumes at the same location. (m)–(q) Otoscopic images acquired during OCT acquisition at five different locations. (f) MIP image of the OCT volume fused from V1 to V5. (l) Corresponding OCT B-scan at the same location with panels (g)–(k).

A representative set of volumes from one patient, following the acquisition protocol described above, is shown in Fig. 3. Figures 3(a)–3(e) show the MIP of OCT volumes at five positions (V1–V5). During OCT acquisition, the corresponding otoscopic images were captured synchronously, as shown in Figs. 3(m)–3(q). The MIP images reveal that OCT volumes acquired at different locations represent portions of the TM and exhibit considerable overlap. In this study, OCT volume 1 (V1) was used as the reference volume, and the others (V2–V5) were registered to V1. After registration, OCT B-scans from volumes in the same position are shown in Figs. 3(g)–3(k). Next, all four registered volumes are fused. The MIP image and corresponding B-scan of the fused OCT volume are shown in Figs. 3(f) and 3(l), respectively. We can see that nearly the entire TM is reconstructed.

**Fig. 4:**
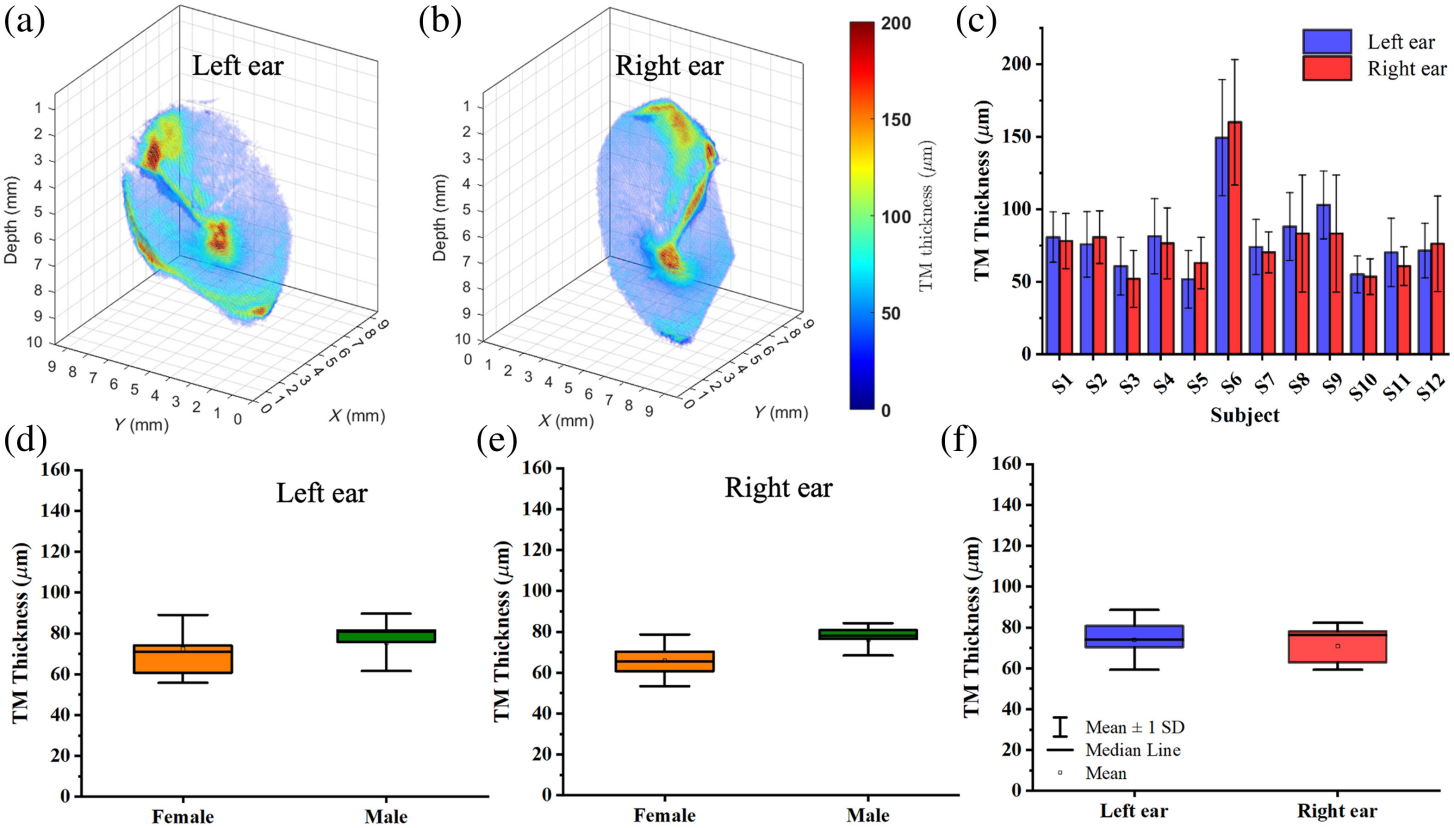
Quantitative analysis of TM thickness comparing left/right and male/female. (a) 3D thickness map of left ear from a normal subject. (b) 3D thickness map of the right ear from the same subject as (a). (c) Quantitative measure of TM thickness between left and right ears in 12 normal subjects. (d) and (e) Boxplots of TM thickness between left and right ears for male and female subjects. (f) Boxplots of combined male and female subject results for left and right ears.

The TM thickness from both the left and right ears of 12 patients was measured using the stitched OCT volumes, as shown in Fig. 4. Representative 3D TM thickness maps from one subject are shown in Figs. 4(a) and 4(b), respectively, where the thickness distribution of the TM can be clearly observed. Figure 4(c) shows the mean and standard deviation of the TM thickness for each of the 12 subjects. Subject 6 exhibited a relatively large TM thickness measurement compared to other subjects. We tested this subject using the interquartile range method and identified subject 6 as an outlier. As a result, we excluded this subject from the subsequent statistical analysis. It is noted that there is no significant difference (p=0.78) in left ear TM thickness between males and females, nor is there a significant difference in right ear TM thickness (p=0.63), as shown in Figs. 4(d) and 4(e). The cumulative (male and female) mean and standard deviation for left ears is 73.89±14.79  μm and for right ears is 70.72±11.58  μm. A statistical comparison of this result, shown graphically in Fig. 4(f), showed no significant difference at 0.05 level.

**Fig. 5:**
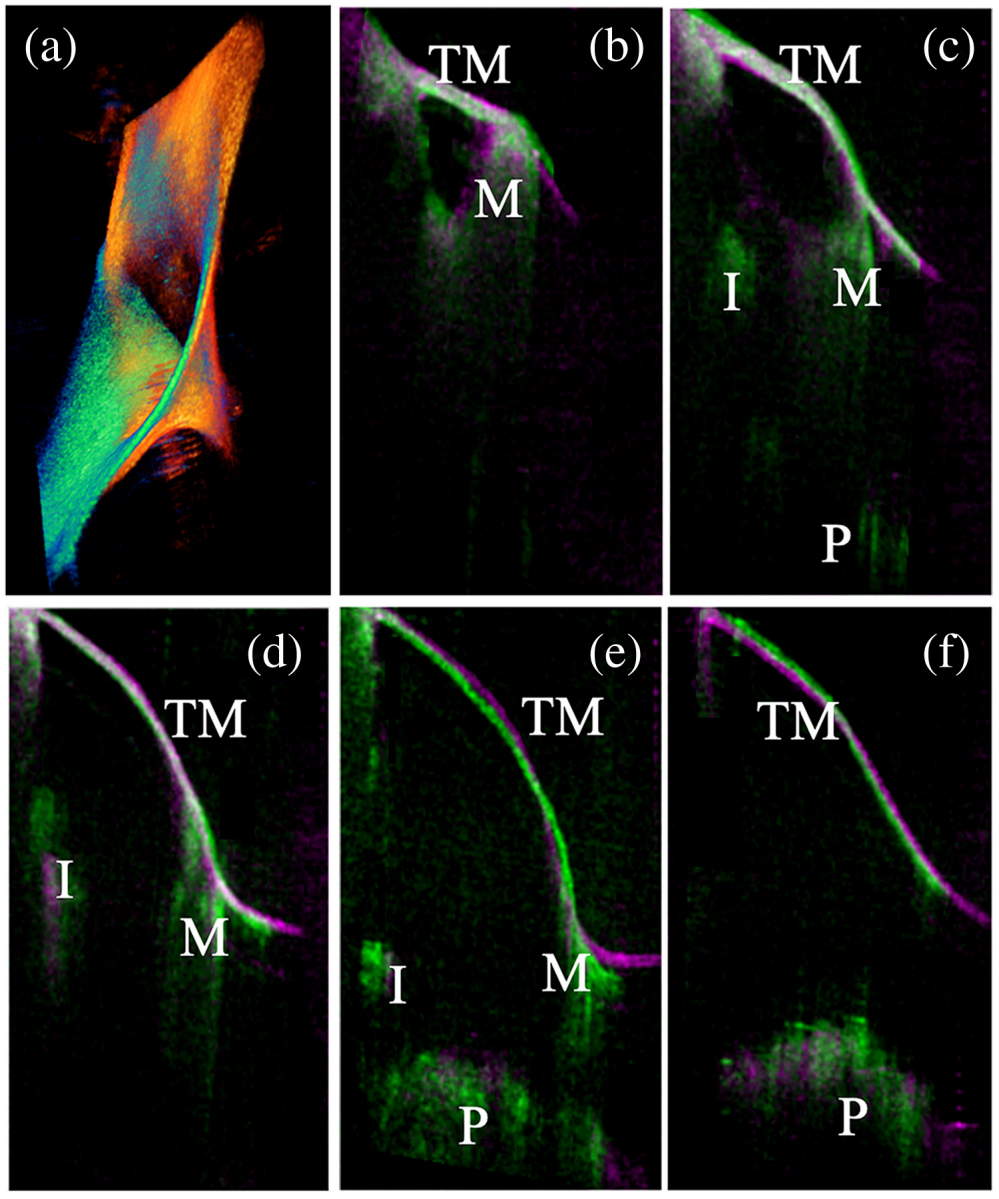
Representative registration results for left and right ear OCT volumes. (a) overlapped OCT volumes, left orange-red, right green-blue. (b)–(f) Representative OCT cross-sections selected from the registered OCT volumes. TM: Tympanic membrane; M: Malleus; I: Incus; U: Umbo; P: Promontory.

To quantitatively evaluate the symmetry of the left and right ears, the OCT volumes of both ears are first carefully registered using the same algorithm used in the volume fusion described above. Figure 5 shows representative registration results for one subject. Figure 5(a) shows the 3D reconstructed registration results, where the left ear is shown in the orange-red colormap, with the right ear depicted in the blue-green colormap. Figures 5(b)–5(f) show consecutive OCT B-scan images from the registered volumes. The left ear is shown in the magenta colormap, with the right ear depicted in the green colormap. Structures from the left and right MEs are well-matched within the cross-sectional images. Qualitatively, the OCT volumes of the left and right ears appear to be well registered.

**Fig. 6:**
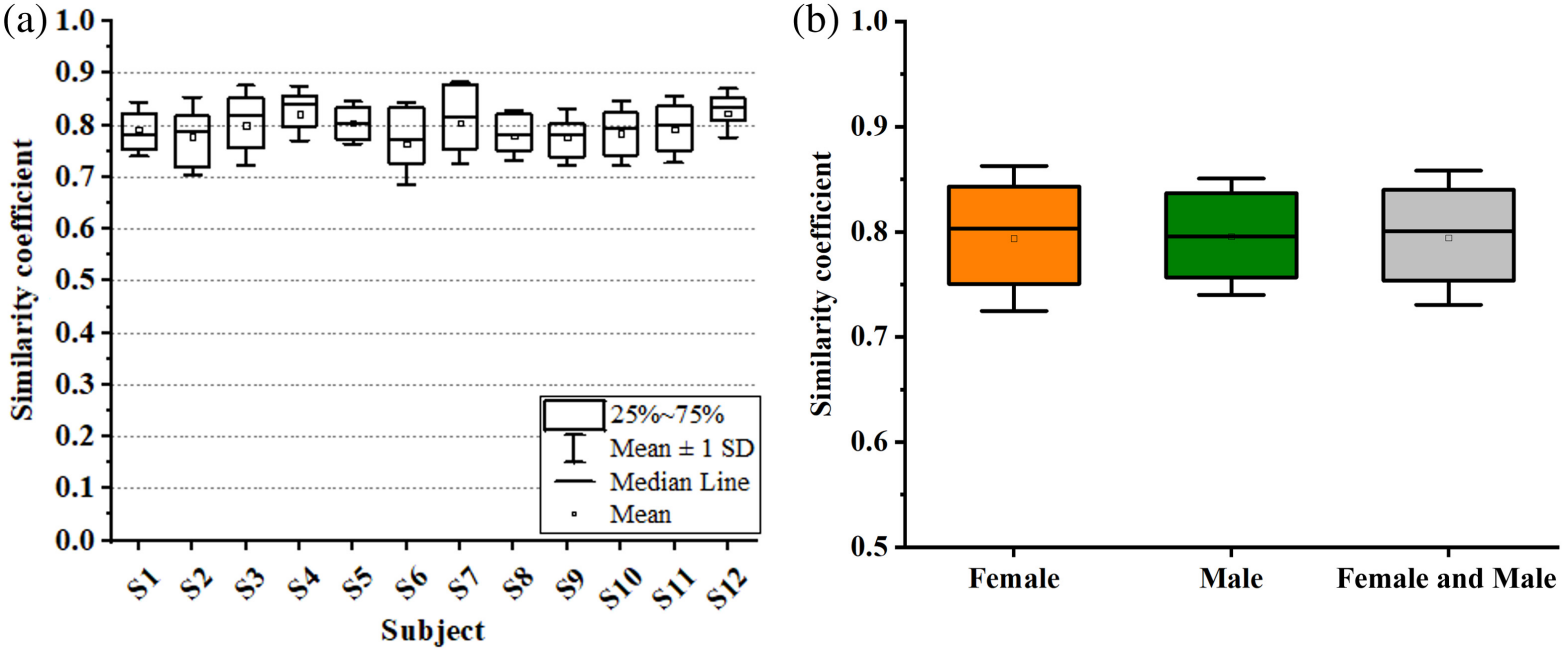
(a) Box plots of the similarity coefficient measured for each subject. (b) Boxplots of cumulative results separated out as female, male, and then combined.

After registering the OCT volumes of the left and right ears, we performed a quantitative analysis of the similarity by application of Eq. (1) to co-registered cross-sections. The mean and standard deviation for each of the 12 normal subjects are shown graphically in Fig. 6. There are no differences between females and males (p=0.73). The cumulative (males and females) mean and standard deviation for all 12 subjects was 0.79±0.02.

## 4 Discussion

Here, we measured the TM thickness and shape in normal human subjects using OCT. We found that there were no differences between ears, nor between patients of different sex. Studies have shown that some ME pathologies are accompanied by changes in TM thickness.^42^ Therefore, we believe that this technique of assessing for differences in TM thickness between a patient’s two ears could be a useful screening tool.

To enlarge our system’s FOV, we developed a registration and stitching algorithm that enabled us to reconstruct a volumetric OCT image encompassing all the TM and ME structures visible through the typical access path of the ear canal. The reconstruction was done in post-processing using a workstation (AMD Ryzen 9 7900X 12-core processor and 64 GB RAM) and software written in MATLAB. Registration of pairs of OCT volumes takes only a few seconds. The entire volume reconstruction process took ∼2  min from start to finish while running the custom GUI program. Consequently, it was not feasible to report the fused volume to the clinician immediately. Going forward, we can dramatically speed this up by rewriting the code in a more efficient language, e.g., C++ or CUDA C. Alternatively, we could increase the FOV of the instrument to reduce the number of volumes needed or eliminate the process altogether. The current limitation to the instrument’s FOV is $7.4\times 7.4\;{\mathrm{mm}}^{2}$. This would decrease the computational burden; however, we would not get the additional benefits of improved SNR and reduced shadowing of structures deep in the ME. The latter is due to the multiple angles the volumes are captured at which necessarily have different shadowing due to strong scatters, e.g., the ossicles.

Subject 6 had no reported prior ME disease; however, it is possible the patient had unreported childhood ear infections that led to some TM thickening. As noted above, subject 6 was identified as an outlier and excluded from the statistical analysis. For completeness, the inclusion of subject 6, who was female, would have increased the mean and standard deviation from 72.47±15.14  μm to 83.45±32.76  μm for the left ear and from 66.05±11.56  μm to 79.46±37.33  μm for the right ear. Even without subject 6, the standard deviation for female subjects was larger than for males. Likewise, the female mean is lower than that of males. A larger study is needed to understand if these relatively small differences persist.

It is also possible that more local differences might prove important for certain pathologies. It is not obvious *a priori* how to subdivide the TM into more local regions that would be relevant. However, we have made the fused OCT volumes, segmented TM images, TM thickness maps, and software for calculating TM thickness available offline so that local TM thickness could be calculated for other researchers interested in these values.

We also examined ear symmetry by quantifying it using the DSC. Intuitively, we expect early signs of ME and/or TM pathology will lead to reduced symmetry. That seems very likely for patients who ultimately develop unilateral disease, but also likely for patients who develop bilateral disease because disease progression will differ on each side. Naturally, to investigate this, we first need to establish the symmetry of normal ears. Our results indicated a high degree of symmetry between the right/left ears with no statistical difference among male/female patients. In this study, we made no effort to ensure that the pressure was equalized within the middle ear. It is possible the DSC would change if we had subjects first perform a Valsalva maneuver to open the Eustachian tube and insufflate the ear.

Understanding the clinical implications of TM size variations is also vital. Changes in TM thickness are associated with several middle ear pathologies, and accurate measurement of these variations could enable more comprehensive diagnoses of various otologic diseases. In future work, we will use this technique to study patients with pathological ears to assess their sensitivity and specificity for detecting disease.

## 5 Conclusion

Early diagnosis of ME and TM pathology is important for improving patient outcomes. However, the current gold standard for otologic clinical diagnostic imaging is still otoscopy, which is subjective and relies heavily on the experience of clinicians. There is a need to develop simple objective quantitative imaging tools that can be used by clinicians during routine office visits to identify early signs of ear pathology. OCT is a noninvasive, high-resolution imaging technology that is rapidly emerging as a potential imaging tool for otology. Here, using OCT volume images, we have established a baseline for the TM thickness in normal ears and the symmetry between the right and left ears. These quantitative measures can serve as the foundation for new diagnostic metrics derived from OCT images for pathologies of the TM and ME.

## Supplementary Material

10.1117/1.JBO.30.5.056007.s01

## Data Availability

De-identified OCT volume images used in this work have been uploaded to Mendeley Data.^44^
