# Supplementary material for: Quantitative measurement of tympanic membrane structure and symmetry with optical coherence tomography in normal human subjects
Source: J Biomed Opt. 2025 May 14;30(5):056007. doi: 10.1117/1.JBO.30.5.056007 (PMC12077914; doi:10.1117/1.JBO.30.5.056007)
Supplement: Supplementary file 1 [file JBO_030_056007_SD001.docx]

**Supplemental Material**

Inside the handheld imaging probe, illustrated in Fig. S1(a), the optical paths for both OCT and video imaging are integrated into a single system. For OCT imaging, a dual-axis MEMS mirror (Mirrocle Technology, UK) scans the OCT beam across the sample by reflecting it through an objective lens (OBJL). For the video imaging path, a high-pass dichroic mirror (DM) positioned between the MEMS mirror and the achromatic objective lens (AL) redirects the back-reflected visible light toward a CMOS sensor (MU9PC-MH, XIMEA, Germany), producing a live video otoscope image. Surrounding LEDs provide illumination of the ear canal, enabling clinicians to use the real-time video feed, displayed on a monitor, for precise positioning of the probe during OCT imaging. Figure S1(b) shows a photo of the complete handheld OCT tower, including a demonstration of the imaging probe being used on a subject's ear.


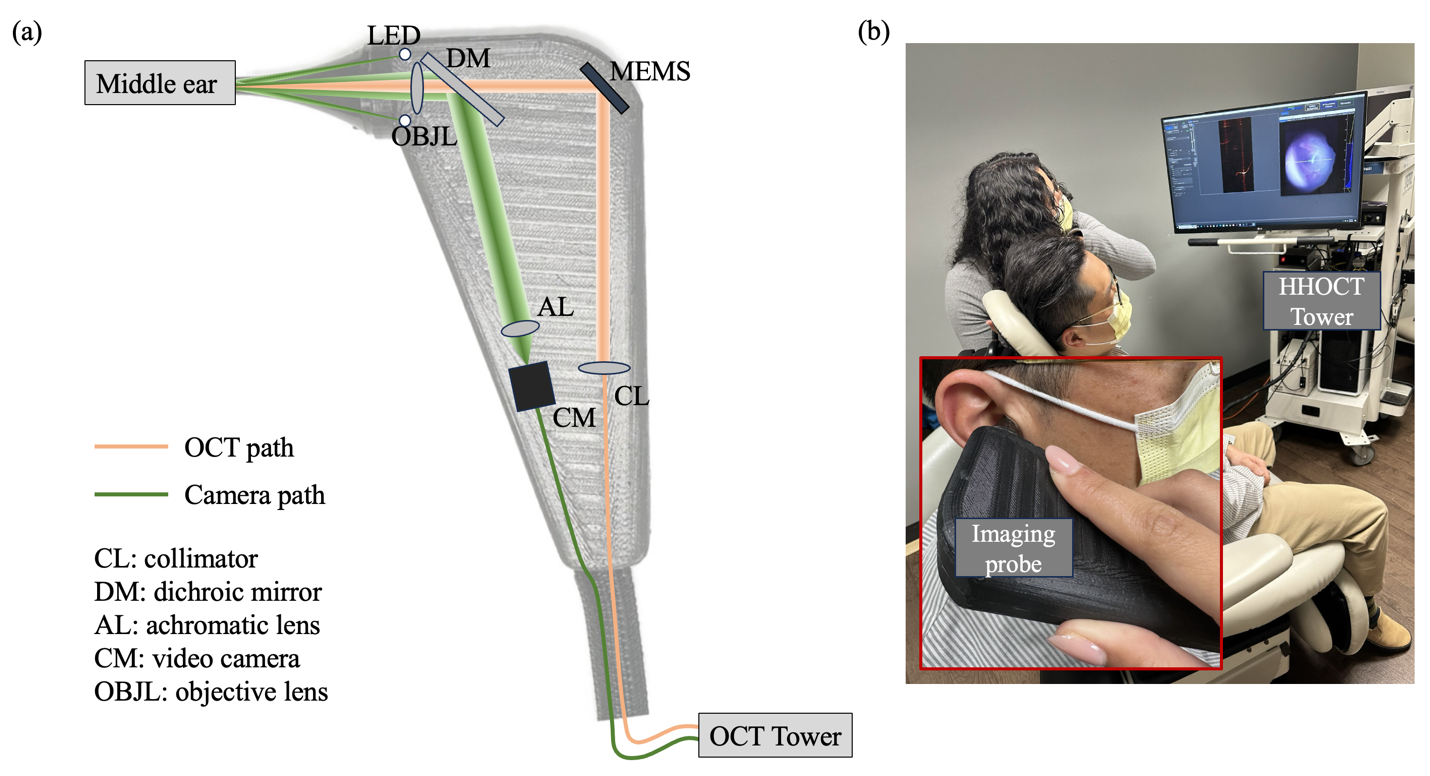


**Figure S1** (a) Schematic diagram of the handheld otologic probe integrating both the OCT and camera optical paths. (b) Handheld OCT (HHOCT) Tower setup on the clinic to image a subject’s ear.

Table S1 presents information on 12 normal subjects (5 males, 7 females) aged 29-84 years. While some had medical histories, none had middle ear abnormalities. OCT imaging was performed on both ears of all subjects.

**Table S1** Information of 12 subjects.

| No. | Sex | Race | Age | Medical History | Ear Exam | Right ear | | | Left ear | | |
| --- | --- | --- | --- | --- | --- | --- | --- | --- | --- | --- | --- |
|  |  |  |  |  |  | PTA | WRS | ABG | PTA | WRS | ABG |
| S1 | M | Asian | 30 | Normal volunteer | - | - | - | - | - | - | - |
| S2 | M | Asian | 29 | Normal volunteer | - | - | - | - | - | - | - |
| S3 | F | Other | 32 | Normal volunteer | - | - | - | - | - | - | - |
| S4 | M | Asian | 84 | SNHL, cochlear implant candidate | Normal bilaterally | 86.67 | 24 | 0 | 61.67 | 32 | 0 |
| S5 | M | White | 57 | Left cochlear schwannoma with SNHL | Normal bilaterally | 20 | 100 | 0 | 116.67 | 0 | 0 |
| S6 | F | White | 72 | Right sudden SNHL | Normal bilaterally | 116.67 | 0 | 0 | 23.33 | 92 | 0 |
| S7 | F | White | 64 | Bilateral tinnitus | Normal bilaterally | 28.33 | 100 | 15 | 11.67 | 100 | 0 |
| S8 | M | Asian | 50 | Eustachian tube dysfunction | Normal bilaterally | 18.33 | 100 | 10 | 16.67 | 100 | 8.33 |
| S9 | F | White | 57 | Bilateral tinnitus | Normal bilaterally | 10 | 100 | 6.67 | 8.33 | 100 | 0 |
| S10 | F | White | 70 | Ear canal ulcerations due to osteonecrosis from osteoporosis medication with normal CT temporal bones | Ear canal ulcerations, normal tympanic membranes bilaterally | 26.67 | 88 | 0 | 25 | 80 | 0 |
| S11 | F | Asian | 63 | Left SNHL, tinnitus | Normal bilaterally | 16.67 | 100 | 0 | 118.33 | 0 | 0 |
| S12 | F | White | 42 | Right single sided deafness s/p cochlear implantation | Normal bilaterally | 105 | 0 | 0 | 20 | 100 | 0 |

SNHL: sensorineural hearing loss, PTA: Pure tone average, WRS: Word recognition score, ABG: Air-bone gap. The 3-frequency PTA using 0.5, 1, and 2 kHz was measured.
